# Radiologic Assessment of Cervical Canal Stenosis Using Kang MRI Grading System: Do Clinical Symptoms Correlate with Imaging Findings?

**DOI:** 10.7759/cureus.5073

**Published:** 2019-07-03

**Authors:** Hira Waheed, Muhammad Salman Khan, Aeman Muneeb, Syed Jahanzeb, Muhammad Nadeem Ahmad

**Affiliations:** 1 Radiology, Aga Khan University Hospital, Karachi, PAK; 2 Internal Medicine, Lincoln Medical Center, New York, USA; 3 Orthopedics, Civil Hospital Karachi, Dow University of Health Sciences, Karachi, PAK

**Keywords:** cervical canal stenosis, spinal cord compression, magnetic resonance imaging

## Abstract

Introduction

Magnetic resonance imaging (MRI) is widely used in the evaluation of cervical canal stenosis and spinal cord compression. Kang et al. formulated a new MRI grading system for assessing canal stenosis which takes cord signal change into account. The purpose of the study was to determine the agreement between Kang's grading system and neurological symptoms.

Methods

A cross-sectional study was conducted at Aga Khan University Hospital between April 2014 and December 2015. Patients meeting inclusion criteria were enrolled. T2 sagittal and T2 axial MRI images were acquired and reported by a consultant neuroradiologist, in accordance with the MRI grading system suggested by Kang et al. Neurologic clinical symptoms were acquired by the history taken by the principal investigator. More than one neurologic symptoms and Kang MRI grade 2 or 3 were taken as positive evidence of cord compression resulting from canal stenosis.

Results

Amongst 126 subjects, 54% were females. Mean age of patients was 50.3 ± 14.3 years (range 19-83 years). Average disease duration was 4.61 ± 3.73 (range 1-24 months). In the majority of the patients, the findings were found at the C5-C6 level. 65.1% of patients were identified positive for cervical canal stenosis by Kang grading system. Most common neurological symptoms were pain (99%) and numbness (56%). Cohen’s Kappa was run to evaluate the agreement between neurological symptoms and Kang grading system. There was a strong agreement between the two methods, K = 0.81 (95% CI 0.70-0.92), p < 0.001.

Conclusion

There was a substantial agreement between Kang's grading system and the presence of clinical symptoms. The agreement was greatest in females, older patients, and those with longer duration of symptoms.

## Introduction

Cervical canal stenosis can result from a multitude of causes and can cause spinal cord compression leading to substantial morbidity. It requires prompt diagnosis and treatment to prevent long-term disabilities secondary to irreversible spinal cord injury [1]. Spinal cord compression affects the cervical cord in 10% cases and its prevalence is reported to be 24.4% [2]. Narrowing of the cervical canal can be caused by several conditions including tumors, infections, trauma, degenerative changes like intervertebral disc herniation, osteophytes, and ossification of posterior longitudinal ligaments [3-5].

The most common presenting symptom is pain; other symptoms include numbness, tingling, weakness, gait instability, bowel and bladder dysfunction, spasticity and paresthesia or rarely, permanent paraplegia [6]. Diagnosis is made with clinical history, neurological signs and radiological investigations including plain radiographs, computed tomography (CT) scans, myelography and magnetic resonance imaging (MRI). MRI is a more sensitive modality than CT scan and is the gold standard for imaging cervical cord [7]. MRI of the spine not only helps in diagnosing but also gives an idea of the possible treatment options [8].

It is important to assess the degree of canal stenosis for the better management of patients. A few studies have described various methods of assessment. Early studies performed by Pavlov et al. [9] and Torg et al. [10] were based on radiographs. Muhle et al. [11] graded cervical canal narrowing according to partial/complete obliteration of the anterior or posterior subarachnoid space, cervical cord compression or displacement. There are, however, a few limitations to this grading system as the definition of partial obliteration was unclear and no consideration was given to the signal change of cord. Signal change of the spinal cords is directly associated with the prognosis and Kang et al. formulated a new MRI grading system taking this parameter into account [3].

A universal criterion would ease the comparison of data from different investigations and would improve communication between clinicians and radiologists. With this purpose in mind, we conducted this study to determine the agreement between Kang's grading system and neurological symptoms.

## Materials and methods

This was a cross-sectional study conducted at the Department of Radiology at Aga Khan University Hospital, Karachi from April 2014 to December 2015 for a total duration of 20 months.

Data sources and study population

Sample Size Calculation 

The sample size was calculated by sample size determination in health studies manual by the World Health Organization. By taking agreement between neurological symptoms and Kang's grading system as 80%, the margin of error 7% and level of confidence 95%, a sample size of 126 subjects was calculated. Through non-probability consecutive sampling, 126 patients were recruited in our study.

Inclusion Criteria 

Adult patients of ages between 18 and 70 years and both sexes referred to the radiology department for MRI cervical spine examination for the evaluation of neurological symptoms.

Exclusion Criteria

Patients who have a history of acute trauma, infections, surgical history, tumors, lumbar spinal stenosis, combined brain infarction or any other intracranial lesion and the patients with symptoms at different cord level were excluded.

Data Collection

Patients meeting the inclusion criteria were enrolled in the study after taking informed consent. T2 sagittal and T2 axial images were taken through the cervical spine on Vantage Titan 3T. MRI reporting for cervical cord compression assessment was performed by a consultant neuroradiologist with five-year clinical experience. The radiologist assessed the presence and grade of cervical cord compression at the maximal narrowing point, in accordance with the MRI grading system suggested by Kang et al. Neurologic clinical symptoms were acquired by the history taken by the principal investigator.

Outcome measures

Based on clinical judgment, more than one neurologic symptom was considered positive evidence of canal stenosis and resulting cord compression. Kang’s MRI grade 2 and above was considered positive evidence of cord compression since grade 0 and 1 stenosis do not involve the spinal cord; while grade 2 (spinal cord deformity) and grade 3 (spinal cord signal change) indicate that the spinal cord has been affected.

Cohen's Kappa statistic was calculated to measure the agreement between MRI grade and clinical symptoms. Statistical significance was set at alpha = 0.05. All statistical analyses were performed using SPSS v. 22.0.

## Results

**Figure 1:**
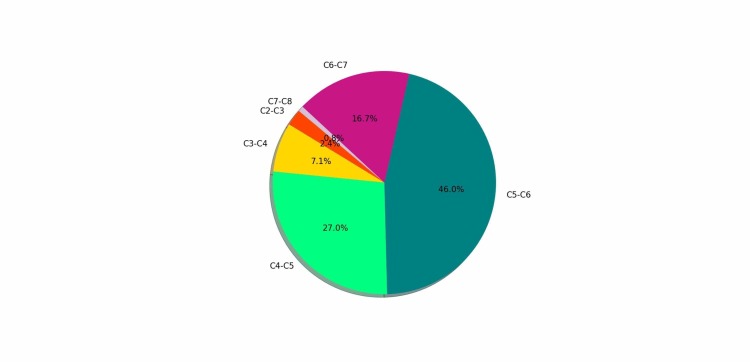
Level of cervical canal stenosis

Amongst 126 subjects, 54% were females. Mean age of patients was 50.3 ± 14.3 years (range 19-83 years). Average disease duration was 4.61 ± 3.73 (range 1-24 months). In the majority of the patients (58/126 or 46.0%), the findings were found at the C5-C6 level (Figure 1).

Pain was the most common clinical symptom followed by numbness and tingling (Table 1)].

**Table 1:** Frequency of neurologic symptoms.

Neurologic Symptoms	Frequency
Pain	125 (99.2%)
Numbness	70 (55.6%)
Tingling	32 (25.4%)
Weakness	5 (4.0%)
Gait Instability	6 (4.8%)

The association between Kang's grading and neurologic symptoms is noted in Table 2. The majority of patients (n = 75, 60%) with any neurologic symptoms had a positive result for cervical stenosis as measured by the Kang's grading system. In addition, kappa statistic revealed a strong agreement between the two methods (K = 0.81, 95% CI: 0.70-0.92, p < 0.001)

**Table 2:** Agreement between Kang's grading system and neurological symptoms.

KANG’S Grading	Neurological Symptoms		Kappa Test	95% CI	p-value
	Positive	Negative			
Positive	75 (59.5%)	7 (5.6%)	0.811	0.704-0.917	p < 0.001
Negative	4 (3.2%)	40 (31.7%)			
Total	79 (62.7%)	47 (37.3%)			

Of note, stratification by gender revealed stronger agreement between symptoms and MRI grading for females compared with males (female: Kappa 0.82; 95% CI: 0.69-0.96 vs. male: Kappa 0.77; 95% CI: 0.58-0.96) (Table 3).

**Table 3:** Gender differences in agreement between Kang's grading system and neurological symptoms of spinal cord compression.

	Neurological Symptoms		Kappa Test	95% CI	p-value
	Positive	Negative			
MALE					
Kang's Positive	41 (70.7%)	4 (6.9%)	0.771	0.582-0.960	p < 0.001
Kang's Negative	1 (1.7%)	12 (20.7%)			
Total	42 (72.4%)	16 (27.6%)			
FEMALE					
Kang's Positive	34 (50%)	3 (4.4%)	0.822	0.686-0.958	p < 0.001
Kang's Negative	3 (4.4%)	28 (41.2%)			
Total	37 (54.4%)	31 (45.6%)			

A sub-analysis stratified by disease duration demonstrated that patients who had been experiencing symptoms for a longer duration (> 5 months) had stronger agreement compared with patients who had been experiencing symptoms for lesser duration (> 5 months: Kappa 0.92; 95% CI 0.76-1.00 vs. < 5 months: Kappa 0.78; 95% CI 0.66-0.91) (Table 4).

**Table 4:** Disease duration differences in agreement between Kang's grading system and neurological symptoms of spinal cord compression.

	Neurological Symptoms		Kappa Test	95% CI	p-value
	Positive	Negative			
LESS THAN 5 MONTHS					
Kang's Positive	55 (56.7%)	7 (7.2%)	0.782	0.655-0.909	p < 0.001
Kang's Negative	3 (3.1%)	32 (33.0%)			
Total	58 (59.8%)	39 (40.2%)			
MORE THAN 5 MONTHS					
Kang's Positive	20 (69%)	0 (0.0%)	0.917	0.757-1.000	p < 0.001
Kang's Negative	1 (3.4%)	8 (27.6%)			
Total	21 (72.4%)	8 (27.6%)			

Stratification by patient age group yielded a Kappa statistic of 0.73 (95% CI 0.53-0.92) for patient age under 55 years and 0.87 (95% CI 0.75-0.99) for patient age over 55 years (Table 5).

**Table 5:** Age differences in agreement between Kang's grading system and neurological symptoms of spinal cord compression.

	Neurological Symptoms		Kappa Test	95% CI	p-value
	Positive	Negative			
LESS THAN 55 YEARS					
Kang's Positive	43 (67.2%)	4 (6.3%)	0.725	0.534-0.915	p < 0.001
Kang's Negative	3 (4.7%)	14 (21.9%)			
Total	46 (71.9%)	18 (28.1%)			
MORE THAN 55 YEARS					
Kang's Positive	32 (51.6%)	3 (4.8%)	0.87	0.747-0.993	p < 0.001
Kang's Negative	1 (1.6%)	26 (41.9%)			
Total	33 (53.2%)	29 (46.8%)			

## Discussion

Cervical canal stenosis can result from a number of causes and lead to cervical spinal cord compression, eventually leading to disability. In this context, multiple image-based grading systems help classify the extent of stenosis and involvement of the spinal cord. They are useful in communicating with clinicians about the extent of the stenosis. Combining radiological grade with clinical examination can build a full picture of the underlying pathology. Multiple systems have been proposed throughout the years, with Kang proposing a new MRI based system [3] that hopes to be more useful clinically. The purpose of our study was to evaluate the agreement and to assess whether this grading system correlates with neurologic symptoms for assessing spinal cord compression, that results from cervical cord stenosis. Among the 126 patients included in the analysis, the majority were middle-aged, females with a mean age of 50 years. Additionally, cervical cord stenosis was noted to occur most frequently at the C5-C6 spinal level. We found a strong agreement, K = 0.81 (95% CI 0.70 - 0.92), p < 0.001 between cervical stenosis causing cord compression (Kang's grade 2 and 3) and clinical symptoms. In addition, stratification analysis based on gender, disease duration, and age, all showed a strong and significant correlation.

Harrop et al. [12] studied cervical spinal cord compression and the presence of hyperintense signal within the cord on T2-weighted imaging. They evaluated the correlation between the radiological findings on MRI cervical spine and cord myelopathy and suggested a close correlation between those radiologic findings and cord myelopathy, but they did not grade the spinal cord compression. Takahashi et al. [13] reported the frequency of the high signal intensity of the cervical cord on T2-weighted imaging is directly proportional to the severity of clinical myelopathy and the degree of spinal canal compression. Similarly, Kang et al. reported a new MRI grading system for cervical canal stenosis. They classified cervical canal stenosis into the following grades based on T2- weighted sagittal images: grade 0, absence of canal stenosis (subarachnoid space obliteration ≤ 50%); grade 1, subarachnoid space obliteration > 50%; grade 2, spinal cord deformity (compressed); and grade 3, spinal cord signal change. Kang et al. suggested that this new grading system provides a reliable assessment of cervical canal stenosis, with the interobserver agreement for the four grades ranging from 0.60 to 0.62 [3]. A similar study by Park et al. [1] reported that most of the patients with grade 0 cervical canal stenosis showed no neurologic manifestation, and patients with grades 2 and 3 cervical canal stenosis had positive neurologic manifestations. The clinical significance of grade 1 cervical canal stenosis was controversial. The agreement between MRI grade and clinical manifestations was high, similar to the findings in the present study.

Kang et al. [3] reported in their study that their grading system has a high interobserver agreement, determined by the intra-class correlation coefficient of 0.768. Park et al. [1] reported an interobserver agreement of 0.925 which was also higher than reported in the study by Kang et al. [3]. Interestingly, our inter-observer agreement was higher than Kang et al. but lower than Park et al. probably due to experimental random error. However, since all studies report substantial reliability, the proposed grading system appears to be an accurate and reliable method for assessing the degree of canal stenosis. Park et al. [1] also reported that clinical correlation was higher in the older age group (over 50 years). Another similar study by Guen et al. [14] reported a new MRI grading system for lumbar canal stenosis. Guen et al. [14] suggested that this new grading system provides a reliable assessment, with the interobserver agreement for four grades ranging from 0.730 to 0.953 (intra-class correlation coefficient reliability). Highest agreement was found at the level of higher grading (K = 0.789), and the agreement for the older age group (55 years) was higher (0.870 vs 0.725) than that for the younger age group (55 years). Female gender and longer duration of disease also had a better agreement. Similarly, our study also showed a better agreement for the older age group [Table 5], longer disease duration [Table 4] and female gender [Table 3].

The results of the study should be interpreted with certain limitations. One such limitation was that our study was based on recumbent MR images due to lack of availability of upright MRI. Studies have shown that conventional recumbent MRI of the cervical spine may underestimate disease because the imaging is performed in a nondynamic, non-weight bearing position.

## Conclusions

In conclusion, Kang et al. grading system show considerable agreement with neurological symptoms of cervical canal stenosis and this agreement is higher in females, older age (>55 years) and those with a longer duration of disease (greater than 5 months). The current study provides useful information to patients, radiologists, and other physicians who may encounter cervical canal stenosis. As such, Kang's grading system can be utilized as a reference criterion to improve communication between clinicians and radiologists.
